# Muscle Oxygen Extraction during Vascular Occlusion Test in Physically Very Active versus Inactive Healthy Men: A Comparative Study

**DOI:** 10.3390/jfmk9020057

**Published:** 2024-03-22

**Authors:** Marcelo Tuesta, Rodrigo Yáñez-Sepúlveda, Matías Monsalves-Álvarez, Aldo Vásquez-Bonilla, Jorge Olivares-Arancibia, Daniel Rojas-Valverde, Ildefonso Alvear-Órdenes

**Affiliations:** 1 Exercise and Rehabilitation Sciences Laboratory, School of Physical Therapy, Faculty of Rehabilitation Sciences, Universidad Andres Bello, Viña del Mar 2520000, Chile; marcelo.tuesta@unab.cl; 2 Laboratory of Sport Sciences, Centro de Medicina Deportiva Sports MD, Viña del Mar 2521156, Chile; 3 Faculty Education and Social Sciences, Universidad Andres Bello, Viña del Mar 2520000, Chile; rodrigo.yanez.s@unab.cl; 4 Instituto de Ciencias de la Salud, Universidad de O’Higgins, Rancagua 2820000, Chile; matias.monsalves@uoh.cl; 5 Faculty of Sport Sciences, Universidad de Extremadura, 10001 Caceres, Spain; alvasquezb@unex.es; 6 Grupo AFySE, Investigación en Actividad Física y Salud Escolar, Escuela de Pedagogía en Educación Física, Facultad de Educación, Universidad de las Américas, Santiago 8320000, Chile; 7 Centro de Investigación y Diagnóstico en Salud y Deporte (CIDISAD-NARS), Escuela Ciencias del Movimiento Humano y Calidad de Vida (CIEMHCAVI), Universidad Nacional, Heredia 86-3000, Costa Rica; drojasv@una.cr; 8 Clínica de Lesiones Deportivas (Rehab&Readapt), Escuela Ciencias del Movimiento Humano y Calidad de Vida (CIEMHCAVI), Universidad Nacional, Heredia 86-3000, Costa Rica; 9 Applied Physiology Laboratory (FISAP), Institute of Biomedicine (IBIOMED), University of León, 24001 León, Spain; ialvor@unileon.es

**Keywords:** near-infrared spectroscopy, physical activity, muscle adaptations

## Abstract

An increase in the delivery and use of oxygen to the musculature in physically active subjects are determinants of improving health-related aerobic capacity. Additional health benefits, such as an increase in the muscle mass and a decrease in fat mass, principally in the legs, could be achieved with weekly global physical activity levels of more than 300 min. The objective was to compare the muscle vascular and metabolic profiles of physically very active and inactive subjects. Twenty healthy men participated in the study; ten were assigned to the physically very active group (25.5 ± 4.2 years; 72.7 ± 8.1 kg; 173.7 ± 7.6 cm) and ten to the physically inactive group (30.0 ± 7.4 years; 74.9 ± 11.8 kg; 173.0 ± 6.4 cm). The level of physical activity was determined by the Global Physical Activity Questionnaire (GPAQ). A resting vascular occlusion test (5 min of an ischemic phase and 3 min of a reperfusion phase) was used, whereas a near-field infrared spectroscopy (NIRS) device was used to evaluate the muscle oxygenation in the right vastus lateralis of the quadriceps muscle. The area under the curve of the deoxyhemoglobin (HHb) during the ischemic phase and above the curve of the tissue saturation index (TSI) during the reperfusion phase were obtained to determine muscle metabolic and vascular responses, respectively. Physically very active group showed a higher absolute HHb (3331.9 ± 995.7 vs. 6182.7 ± 1632.5 mmol/s) and lower TSI (7615.0 ± 1111.9 vs. 5420.0 ± 781.4 %/s) and relative to body weight (46.3 ± 14.6 vs. 84.4 ± 27.1 mmol/s/kg and 106.0 ± 20.6 vs. 73.6 ± 13.8 %/s/kg, respectively), muscle mass (369.9 ± 122.2 vs. 707.5 ± 225.8 mmol/kg and 829.7 ± 163.4 vs. 611.9 ± 154.2 %/s/kg) and fat mass (1760.8 ± 522.9 vs. 2981.0 ± 1239.9 mmol/s/kg and 4160.0 ± 1257.3 vs. ±2638.4 ± 994.3 %/s/kg, respectively) than physically inactive subjects. A negative correlation was observed between HHb levels and TSI (r = −0.6; *p* < 0.05). Physically very active men (>300 min/week) present better muscle oxidative metabolism and perfusion and perform significantly more physical activity than physically inactive subjects. Extra benefits for vascular health and muscle oxidative metabolism are achieved when a subject becomes physically very active, as recommended by the World Health Organization. In addition, a higher level of physical activity determined by GPAQ is related to better vascular function and oxidative metabolism of the main locomotor musculature, i.e., the quadriceps.

## 1. Introduction

Physical activity (PA) is an important tool in health promotion and prevention. Therefore, physically inactive subjects are at greater risk of suffering from chronic noncommunicable diseases such as hypertension, diabetes, metabolic syndrome, and arthrosis [1,2]. Conversely, adult subjects who are physically active (i.e., 150 to 300 min of moderate or 75 to 150 min of vigorous physical activity) reduce the risk of developing/aggravating these diseases or of dying from cardiovascular disease or other causes [3]. Additional health benefits in adults have been highlighted when moderate-to-vigorous physical activity (MVPA) is more than 300 min per week [4]. One of the most important effects of achieving these benefits is the increase in aerobic capacity (i.e., the increase in maximal oxygen uptake) [5]. Two determining factors to increase aerobic capacity are the adequate delivery of oxygen to the active muscles (i.e., local vascular response) and the extraction capacity of the muscles (i.e., oxidative metabolism) [6]. Both have been previously studied using near-field infrared spectroscopy (NIRS). NIRS is a non-invasive technology capable of assessing the muscle´s chromophores (i.e., oxygenated hemoglobin or oxyhemoglobin [O_2_Hb], deoxygenated hemoglobin or deoxyhemoglobin [HHb] and total hemoglobin) through the attenuation of light as it passes through the tissues [7]. The tissue saturation index [TSI] depends on [O_2_Hb] and [HHb], as follows: TSI = [O_2_Hb]/([O_2_Hb] + [HHb])*100%. This last index has been proposed as an indicator of muscle oxygenation. Both [HHb] and TSI analyzes have been used, in subjects at rest, in vascular occlusion tests (VOT) with an ischemic phase due to arterial occlusion (e.g., ~5 min) and a subsequent phase of reperfusion (~3–5 min). This procedure has been used to estimate both skeletal muscle oxidative metabolism (using the HHb value) and peripheral muscle blood flow (using the TSI value) [6]. Thus, muscle oxygen consumption in the muscle with NIRS could reflect the cardiorespiratory benefits induced by elevated regular physical activity in non-athletes, which could be related to greater changes in muscle fractional oxygen extraction. However, this relationship is still unknown.

High levels of physical activity are expected to be associated with greater changes in body composition, with greater muscle mass and lower fat mass. These adaptations have been related to better cardiometabolic health in physically active individuals with high cardiorespiratory fitness [8]. However, the measurement of muscle oxygen extraction may represent a simpler-to-apply and lower-cost method for the study of health-related fitness than the measurement of pulmonary oxygen consumption. Moreover, this assessment can be applied in less rigorous laboratory or field conditions than the cardiopulmonary exercise test for measuring pulmonary oxygen consumption. Based on the above, it is highly relevant to analyze the muscle metabolism and perfusion (blood flow) capacities of physically active subjects. Therefore, the aim of the study was to compare the muscle vascular and oxidative metabolic profiles of physically very active (i.e., >300 min/week) and non-physically active subjects.

## 2. Materials and Methods

### 2.1. Type of Study

Non-experimental descriptive study of cross-sectional design with a correlational-causal approach.

### 2.2. Participants

A purposive sample of 20 healthy men physically active with physical activity levels more than 300 min per week (PA; *n* = 10; age = 30.0 ± 7.4 years) and physically inactive (NPA; *n* = 10; age = 25.5 ± 4.2 years) subjects were recruited from the active and administrative service departments, respectively, belonging to a military unit of the Valparaíso region, Chile. The military institution promotes 3 classes/week (~1 h) of physical training for the improvement of general physical capacity (i.e., aerobic exercise, strength, and stretching) and 3 days (~1.5 h/d) of military exercises, (i.e., military operations and use of war material) for active service subjects. However, administration department subjects promote 2 classes/week (~1 h) of physical training for general physical capacity improvement. Participants were included if they had not taken any drugs or nutritional supplements for at least 6 months prior to the study. In addition, participants with a recent (<6 months) moderate to severe musculoskeletal injury or musculoskeletal sequela, neurological disorder, or cardiovascular impairment that may have altered physical activity or inactivity were excluded as appropriate. All enrolled subjects were able to participate in the study.

All participants voluntarily signed an informed consent form detailing the objectives and procedures of the study. A meeting was also held to discuss the procedures and implications of the study. The protocol and development of the present study were approved by the Scientific Ethics Committee of the Universidad Viña del Mar (Code 62-19R), and the current guidelines of the Helsinki Declaration for research on human beings were considered [9]. 

### 2.3. Protocol

To carry out the procedures, participants were summoned only once to the laboratory. In order, a general kinesiological assessment was performed first (exclusion criteria were ruled out). Then, a questionnaire was carried out to assess the level of physical activity (see Section 3.2). Once the questionnaire had been completed, body composition parameters were measured using bioimpedance measurements. Finally, the subject was placed on a stretcher to measure muscle oxygenation parameters (muscle metabolism and blood flow changes) during a vascular occlusion test. Prior to the measurements, participants were asked to refrain from consuming alcohol, caffeine, or any cardiovascular system stimulant supplements for 24 h prior to the day of evaluation. Procedural conditions were set at a temperature of approximately 22–23 °C, while external stimuli, e.g., ambient noise, body movement, among others, were minimized.

## 3. Measurements

### 3.1. Body Composition

Muscle mass and fat mass of the body and legs were assessed using an octopolar electrical bioimpedance or BIA device (model 230, Inbody^®^, Cerritos, CA, USA). All participants were evaluated between 07:00 and 09:00 hours. They were asked to abstain from caffeine and alcohol consumption and from intense physical exercise within 24 h prior to the measurements. In addition, they were asked to arrive well-hydrated and not apply any type of cosmetic or medical product to the skin, such as lotions or ointments [10]. Participants who had any metallic material (e.g., osteosynthesis, catheter, etc.) were discarded.

### 3.2. Physical Activity Level

To evaluate the level of physical activity, the Global Physical Activity Questionnaire (GPAQ) was used. GPAQ is a simple method that allows, among others, to evaluate MVPA levels in people between 15 and 69 years old. It is a questionnaire recommended by the World Health Organization [WHO] as part of a step-by-step strategy to monitor risk factors for chronic diseases [10]. Then, participants were classified as physically very active if they performed more than 300 min of moderate to vigorous intensity physical activity during a week, or physically inactive when they did not achieve at least 150 min of moderate to vigorous intensity physical activity [3].

### 3.3. Muscle Metabolism and Vascular Response

To assess muscle metabolism and the blood flow changes in the quadriceps during VOT, a NIRS device (Portamon, Artinis Medical Systems^®^, Elst, The Netherlands) was used. The NIRS was placed on the vastus lateralis quadriceps of the non-dominant leg, 15 cm above the superior border of the patella and 5 cm lateral to the midline of the thigh in the direction of the muscle fibers. To choose the dominant leg, the subject was asked the following question: If you had to throw a ball at a target, which leg would you use to throw the ball? The one not chosen would be the non-dominant one.

Prior to NIRS placement, the skin had been shaved and cleaned with 70% isopropyl alcohol. The NIRS device was fixed with dermal adhesive tape, and during the evaluation, the limb was covered with a black cloth to avoid ambient light interference with the analyzed signal. The skinfold thickness was assessed with a skinfold meter (Harpenden^®^, London, UK). Subsequently, participants were placed in a supine decubitus position on a clinical stretcher. Fractional muscle oxygen extraction was assessed during a VOT lasting 12 min in total, where the last 2 min of the initial 10 min rest period (i.e., 10 min) were used to determine the basal level of HHb and TSI. A cuff was then inflated to ~240 mmHg in the most distal thigh area, generating ischemia for 5 min to measure muscle metabolism through changes in the area under the HHb curve [11]. 

Once 5 min had elapsed, the cuff was released and 3 min of reperfusion were used to analyze vascular response through changes in TSI [2,12]. For all measurements, NIRS was set to a sampling rate of 10 Hz. Both [HHb] and %TSI used for analyses were obtained from the emitter at 40 mm, as this distance allows greater penetration into muscle tissue [13]. The area under the [HHb] curve corresponded to the entire area above the micromolar concentration variation of the [HHb] line from cuff inflation for 5 min. The area above the TSI curve expressed as percentage variation was obtained from the onset of cuff pressure release (just after 5 min of occlusion). For both variables, the baseline value represented no variation.

## 4. Statistical Analysis

The mean and standard deviation statistics were used to present the variables. The normality of the data distribution (Shapiro–Wilk test) was tested first. An *t*-test was applied to compare the results obtained from the groups. To identify the association between muscle vascular and oxidative metabolism in the participants, a Pearson’s correlation test was used. A *p*-value < 0.05 was considered significant for all tests. GraphPad Prism^®^, La Jolla, CA, USA, version 7.0 software was used for statistical analyses.

## 5. Results

Table 1 shows the demographic and anthropometric characteristics of the participants, i.e., age, anthropometry measurements, and physical activity habits. Here, only the level of weekly physical activity (NPA: 117.3 ± 18.1 vs. PA: 547.3 ± 31.1 min/week) and the skinfold thigh (NPA: 15.6 ± 4.6 vs. PA: 11.1 ± 2.4 mm) between both groups were significantly different with *p* < 0.05.

**Figure 1:**
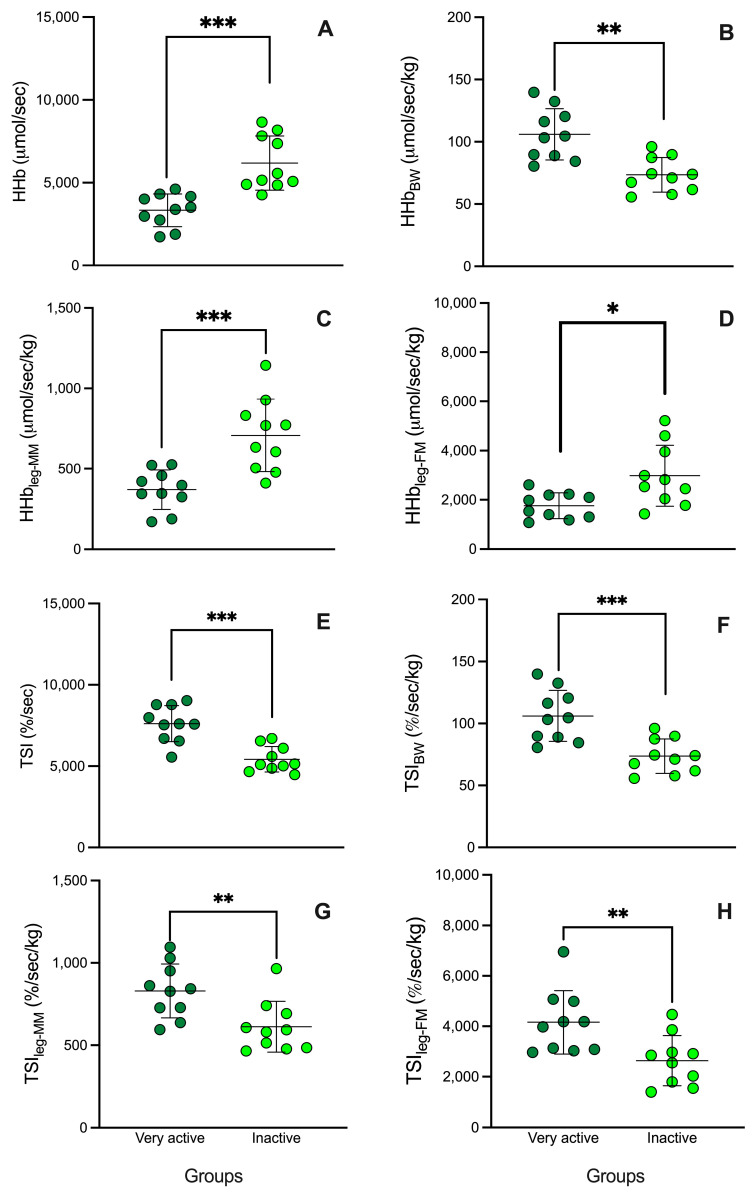
HHb and TSI response during vascular occlusion test in very active (dark green) and inactive participants (light green). BW: Body weight, MM: muscle mass, and FM: fat mass. * Significant differences between groups (*p*-value: * < 0.05, ** < 0.01, *** < 0.001).

Regarding the main muscle oxygenation variables, Figure 1 shows that absolute (3331.9 ± 995.7 vs. 6182.7 ± 1632.5 mmol/s [Figure 1A]) and relatives [HHb] (to total body weight: 46.3 ± 14.6 vs. 84.4 ± 27.1 mmol/s/kg [Figure 1B], leg muscle mass: 369.9 ± 122.2 vs. 707.5 ± 225.8 mmol/kg [Figure 1C], and leg fat mass: 1760.8 ± 522.9 vs. 2981.0 ± 1239.9 mmol/s/kg [Figure 1D]) during occlusion phase were lower in the physically active group than the inactive, respectively (*p* < 0.05). However, during reperfusion phase, absolute (7615.0 ± 1111.9 vs. 5420.0 ± 781.4 %/s [Figure 1A]) and relatives TSI (to body weight: 106.0 ± 20.6 vs. 73.6 ± 13.8 %/s/kg [Figure 1B]), leg muscle mass (829.7 ± 163.4 vs. 611.9 ± 154.2 %/s/kg [Figure 1C]), and leg fat mass (4160.0 ± 1257.3 vs. ±2638.4 ± 994.3 %/s/kg [Figure 1D) showed a higher in the physically active group than the inactive, respectively (*p* < 0.05).

**Figure 2:**
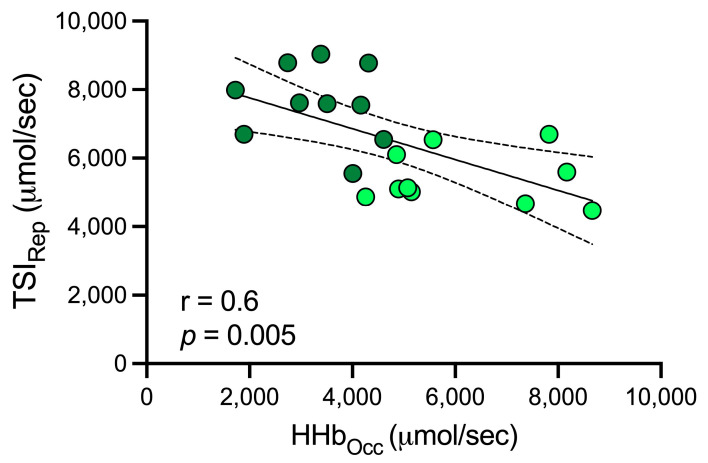
Correlation between TSI and HHb concentrations. TSI_Rep_: tissue saturation index during reperfusion; HHb_Occ_: deoxyhemoglobin during occlusion. Dark green circles = physically very active and Light green circles = physically inactive. Significant differences with *p*-value < 0.05.

Figure 2 showed a high negative correlation was observed between HHb levels (during occlusion) and TSI (during reperfusion) (r = 0.6; *p* < 0.05).

## 6. Discussion

This study compared the oxidative metabolism and vascular function of the vastus lateralis quadriceps muscle between physically very active (>300 min/week) and inactive subjects (<150 min/week) using near-field infrared spectroscopy during a vascular occlusion test. Our main results showed that subjects performing high weekly levels of moderate to vigorous physical activity (~547.3 min/week) measured with GPAQ had a higher oxidative metabolic efficiency during occlusion and microvascular response during reperfusion in quadriceps muscle than physically inactive subjects (~117.3 min/week).

A determining factor in the increase in oxidative metabolism is the muscular oxygen extraction capacity (i.e., arteriovenous oxygen difference) [14]. Previous studies have confirmed that changes in HHb levels measured by NIRS during an occlusion are able to interpret what happens to the muscular oxygen extraction capacity [15]. In trained healthy subjects, lower [HHb] have been observed during arterial occlusion than in untrained subjects, indicating a higher oxidative metabolic capacity of the former [16]. In our study, the same thing happened with the subjects who were classified by the GPAQ as more physically active. This means that the most physically active subjects are more efficient in activating the aerobic-anaerobic transition (i.e., low [HHb]), typical of healthier subjects with good physical fitness. Therefore, the questionnaire (GPAQ) can allow collecting the necessary information for prevention policies for chronic noncommunicable diseases, such as cancer, cardiovascular diseases, and diabetes. mellitus [1,17]. According to the WHO, more than 300 min/week of regular physical activity cause additional effects on people’s health, which guarantees control over the prevention of chronic noncommunicable diseases [4]. In our study, higher levels of physical activity (i.e., ~500–550 min/week) measured with the GPAQ were related to higher values of oxidative capacity (Figure 2), demonstrating that the GPAQ can be a tool sensitive to measure health-oriented physical activity and, indirectly, physiological peripheral adaptations of muscle oxidative capacity in physically very active subjects. This association was also observed when the variables were adjusted for body weight, leg-muscle mass, and leg-fat mass in the studied non-dominant leg. Therefore, a higher oxidative metabolic activity of the main locomotor muscle was associated with healthier morphological adaptations induced by exercise, such as decreasing body weight and leg fat mass, and increasing leg muscle mass. These adaptations have also been linked to the slowing of aging-induced functional decline [18]. Previously, Lagerwaard et al. [19] demonstrated that NIRS is a suitable method to measure the effects of aging on mitochondrial function in locomotor muscles, in particular the quadriceps, of healthy subjects. In this regard, a higher fractional oxygen extraction in the physically very active group than the inactive group during both stages of VOT (i.e., ischemia and reperfusion) could provide important protection against the deterioration of muscle function.

However, during reperfusion (in VOT), NIRS has allowed us to assess the context of microvascular function through TSI analysis [20]. Previous conditions of ischemia promote vasodilator metabolite productions (i.e., hydrogen ions, adenosine diphosphate, etc.), which dilate arterioles and decrease vascular resistance to increase oxygen delivery and tissue protection. When disocclusion of the cuff occurs, blood flow suddenly increases in the microvasculature (reperfusion), accelerating the muscle oxygen restoration rate and increasing the TSI [21,22]. It is recognized that regular physical activity in healthy subjects increases the microvascular response (endothelial function) [23], which becomes a vascular protective factor against the occurrence of cardiovascular risk factors such as dyslipidemia, hyperglycemia, and hypertension, among others [24]. Muscle adaptations induced by high levels of physical activity such as angiogenesis, decreased peripheral vascular resistance, increased muscle mass, and increased mitochondrial density have been related to increased muscle oxygenation and better health status (fewer cardiovascular and metabolic risk factors) [25,26,27]. In physically active subjects, a higher TSI has been observed than in inactive participants during reperfusion [28], as occurred in our studies with the absolute and relative TSI of physically very active subjects compared to the non-active. In this group, higher levels of TSI during reperfusion allow a more rapid activation of aerobic metabolism, favoring their recovery. Higher levels of TSI at reperfusion have been associated with high levels of physical fitness, young age [21], male sex [28], low body fat [29], and high maximal pulmonary oxygen uptake [30]. The latter is the main indicator of health-oriented physical fitness (increased survival) [31]. On the contrary, worse TSI levels are observed in all cardiovascular and metabolic diseases, with an elevated probability of morbi-mortality [32,33]. 

Likewise, the association observed between HHb and TSI (Figure 2) shows that healthy subjects with higher vascular function during reperfusion probably had a higher oxidative capacity during occlusion, demonstrating the importance of vascular adaptation (increased oxygen supply) to increase muscle oxygen extraction and utilization capacity. We think more studies relating muscle oxygenation adaptations together with health monitoring indicators (i.e., high blood pressure, lipids, and/or glycemia) in groups with different levels of physical activity measured with GPAQ, ideally with a controlled and longitudinal design, are needed. In this way, VOT could be used in the future by health professionals to assess the progress of clinical goals of increasing health-oriented physical activity. 

### Limitations

A limitation of the study was the low number of participants; therefore, the results should be viewed with caution. Regarding the use of NIRS, it is recognized that the signal is affected by the adipose tissue present at the measurement site. In the group of physically inactive subjects, the value was higher than the active ones; however, both values were lower than recommended for the skinfold thigh (i.e., 20 mm) [7]. Another factor associated with possible changes in the NIRS reading is the typological composition of the thigh in the measured area, which could vary between very active subjects with respect to non-active ones. 

## 7. Conclusions

In summary, significantly better muscle oxidative metabolism and perfusion were identified in physically very active subjects (>300 min/week), who perform significant more physical activity than physically inactive subjects. Extra benefits on vascular health and muscle oxidative metabolism are achieved when a subject becomes physically very active, as recommended by the WHO. Also, our data support that the higher level of physical activity as determined by GPAQ is related to better vascular function and oxidative metabolism of the main locomotor musculature, i.e., the quadriceps. GPAQ becomes a reliable tool for experts in pre-health prevention through the control of physical activity in the at-risk population.

## Figures and Tables

**Table 1:** Demographic and anthropometric variables of the study participants.

Variables	Active (*n* = 10)	Inactive (*n* = 10)	*p*-Value
Age (years)	30.0 ± 7.4	25.5 ± 4.2	0.11
Weight (kg)	72.7 ± 8.1	74.9 ± 11.8	0.63
Height (cm)	173.7 ± 7.6	173.0 ± 6.4	0.83
Body mass index (kg/m^2^)	24.0 ± 1.4	24.9 ± 2.9	0.45
Fat mass (kg)	12.9 ± 3.9	15.6 ± 6.5	0.28
Muscle mass (kg)	34.2 ± 3.5	33.7 ± 6.2	0.85
Non-dominant leg fat mass (kg)	1.9 ± 0.47	2.3 ± 0.88	0.29
Non-dominant leg muscle mass (kg)	9.0 ± 1.4	9.2 ± 1.0	0.75
MVPA (min/week)	547.3 ± 31.1 *	117.3 ± 18.1	<0.01
Skinfold thigh (mm)	11.1 ± 2.4 *	15.6 ± 4.6	0.01

MVPA: Moderate-vigorous physical activity. * Significant differences between groups.

## Data Availability

The data of this study are available upon request from the corresponding author.
